# White noise speech illusion and psychosis expression: An experimental investigation of psychosis liability

**DOI:** 10.1371/journal.pone.0183695

**Published:** 2017-08-23

**Authors:** Lotta-Katrin Pries, Sinan Guloksuz, Claudia Menne-Lothmann, Jeroen Decoster, Ruud van Winkel, Dina Collip, Philippe Delespaul, Marc De Hert, Catherine Derom, Evert Thiery, Nele Jacobs, Marieke Wichers, Claudia J. P. Simons, Bart P. F. Rutten, Jim van Os

**Affiliations:** 1 Department of Psychiatry and Psychology, Maastricht University Medical Centre, Maastricht, The Netherlands; 2 Department of Psychiatry, Yale School of Medicine, New Haven, Connecticut, United States of America; 3 University Psychiatric Centre KU Leuven, Leuven, Belgium; 4 Centre of Human Genetics, University Hospitals Leuven, KU Leuven, Leuven, Belgium; 5 Department of Obstetrics and Gynecology, Ghent University Hospitals, Ghent University, Ghent, Belgium; 6 Department of Neurology, Ghent University Hospital, Ghent University, Ghent, Belgium; 7 Faculty of Psychology and Educational Sciences, Open University of the Netherlands, Heerlen, The Netherlands; 8 University of Groningen, University Medical Center Groningen, Department of Psychiatry, Interdisciplinary Center Psychopathology and Emotion regulation (ICPE), Groningen, The Netherlands; 9 GGzE, Institute for Mental Health Care Eindhoven and De Kempen, Eindhoven, The Netherlands; 10 King's College London, King's Health Partners, Department of Psychosis Studies, Institute of Psychiatry, London, United Kingdom; United (Osaka U, Kanazawa U, Hamamatsu U Sch Med, Chiba U and Fukui U) Graduate School of Child Developmen, JAPAN

## Abstract

**Background:**

An association between white noise speech illusion and psychotic symptoms has been reported in patients and their relatives. This supports the theory that bottom-up and top-down perceptual processes are involved in the mechanisms underlying perceptual abnormalities. However, findings in nonclinical populations have been conflicting.

**Objectives:**

The aim of this study was to examine the association between white noise speech illusion and subclinical expression of psychotic symptoms in a nonclinical sample. Findings were compared to previous results to investigate potential methodology dependent differences.

**Methods:**

In a general population adolescent and young adult twin sample (n = 704), the association between white noise speech illusion and subclinical psychotic experiences, using the Structured Interview for Schizotypy—Revised (SIS-R) and the Community Assessment of Psychic Experiences (CAPE), was analyzed using multilevel logistic regression analyses.

**Results:**

Perception of any white noise speech illusion was not associated with either positive or negative schizotypy in the general population twin sample, using the method by Galdos et al. (2011) (positive: ORadjusted: 0.82, 95% CI: 0.6–1.12, *p* = 0.217; negative: ORadjusted: 0.75, 95% CI: 0.56–1.02, *p* = 0.065) and the method by Catalan et al. (2014) (positive: ORadjusted: 1.11, 95% CI: 0.79–1.57, *p* = 0.557). No association was found between CAPE scores and speech illusion (ORadjusted: 1.25, 95% CI: 0.88–1.79, *p* = 0.220). For the Catalan et al. (2014) but not the Galdos et al. (2011) method, a negative association was apparent between positive schizotypy and speech illusion with positive or negative affective valence (ORadjusted: 0.44, 95% CI: 0.24–0.81, *p* = 0.008).

**Conclusion:**

Contrary to findings in clinical populations, white noise speech illusion may not be associated with psychosis proneness in nonclinical populations.

## Introduction

Epidemiological data indicate that subtle expression of psychosis—in the form of attenuated reality distortion, such as over-valued ideas and perceptual disturbances—is prevalent across the general population, with some predictive value for subsequent mental disorders [1, 2]. Recent findings from genetic [3] and environmental studies of psychosis [4, 5] also suggest etiological continuity across different levels of psychosis manifestation. In this regard, it is reasonable to argue that both subtle psychotic experiences (e.g., perceptual disturbances and rare illusion) and clinically relevant psychotic symptoms (e.g., disturbing hallucinations) may originate from converging neurocognitive processes.

Emerging neuropsychological theories argue that hallucinations might indeed result from an imbalance between top-down (internal factors, e.g., perceptual expectations, prior knowledge, and mental imagery) and bottom-up (i.e., external sensory input) perceptual processing [6]. Top-down processing may have a substantial impact on experiences, for instance, during the identification of degraded or half visible objects. Even more so, during the formation of hallucinations, top-down processing is thought to activate a percept with no external stimulus. In other words, hallucinations are formed when the final percept is disproportionally influenced by internal factors, thus, when top-down processing is prioritized [7, 8].

Previous studies found that exposure to white noise (sound fragments of random noise) induces the perception of speech illusion, presumably by increasing perceptual expectations, thus, operating on top-down processing [6, 9]. Several studies observed higher prevalence rates of white noise speech illusion in patients with psychosis and their relatives compared to control cases: around 33% in patients with psychotic disorders, 14% in healthy relatives of patients, and 9% in healthy controls [10–12]. Furthermore, positive psychotic experiences correlated with increased rates of white noise speech illusion in patients and their relatives [11]. Thus, in general, these findings support the notion that the white noise task can be used to understand hallucinations within the framework of top-down processing.

The question rises whether the white noise task can also be used to study perceptual abnormalities in nonclinical samples and whether similar mechanisms (i.e., top-down and bottom-up imbalance) are at play in patients, their relatives, and in healthy controls. Studies using the white noise task to investigate the association between speech illusion and psychotic experiences in the general population are scarce and their findings contradict each other. Several studies found a positive association between subclinical psychotic experiences and white noise speech illusion. Randell et al. [13], for instance, investigated white noise speech illusion in undergraduate students and found that participants with higher scores on unusual experiences perceived more speech illusion, especially of abstract nature (words like myth, abyss, and sorrow) rather than of concrete nature (words like desk, arm, and letter). Furthermore, Galdos et al. [11] found a positive association between positive schizotypy and speech illusion in the white noise task. However, there are also several studies that did not find the above-mentioned association. Using the same measurement tools as Galdos et al. [11], Catalan et al. [10] observed no association between positive schizotypy and speech illusion in a nonclinical population. Furthermore, in a recent study in healthy children, the experience of hallucinations (last month) and negative affect (last month and lifetime) were only associated with speech illusion in the white noise task when the focus was directed to the affectively salient illusion variable [14].

Inconsistent results may be partly explained by differences between methodologies, such as measurement tools for psychopathology, sample characteristics, cut-off values for the dichotomous speech illusion variable and analytical strategies [10, 11, 13, 14]. Deeper understanding of underlying mechanisms of perceptual abnormalities in nonclinical populations may foster scientific advances by helping us appraise the dimensional nature and phenomenological features of hallucinations. Therefore, the verification of reliable assessment tools is essential, all the more when the literature is divided. In the current study, we aim to investigate the link between white noise speech illusion and positive subclinical experiences by replicating the various methodologies of preceding studies of the white noise task in the general population [10, 11].

## Methods

Participants were recruited from the East Flanders Prospective Twin Survey (EFPTS; [15]), a prospective population-based, multi-birth registry situated in Flanders, Belgium. Zygosity was determined through sequential analysis based on sex, fetal membranes, umbilical cord blood groups, placental alkaline phosphatase, and DNA fingerprints (EFPTS; [15]). Individuals who were registered in the EFPTS and who fulfilled the inclusion criteria were invited to participate in the TwinssCan project, a longitudinal study collecting data on adolescents and young adults between the ages of 15 and 35 years, including twins, their siblings, and parents. The TwinssCan project, which started enrollment in April 2010, is a general population based, ongoing longitudinal study [16] and currently contains information on 1116 participants; twins (n = 830), siblings (n = 43), and parents (n = 234). Participants were included if they clearly understood the study procedure and were able to provide valid, reliable, and complete data. All participants gave written informed consent; for participants below the age of 18, parent(s) also signed an informed consent. Participants were excluded if they had a pervasive mental disorder as indicated by caregivers. The local ethics committee (Commissie Medische Ethiek van de Universitaire ziekenhuizen KU Leuven, Nr. B32220107766) approved the study. The current study used a subpopulation of the TwinssCan (n = 704) including only monozygotic (MZ) and dizygotic (DZ) twin pairs with complete data on SIS-R, the white noise task, age and sex. Table 1 reports the overview of the demographic variables.

**Table 1:** Summary of selected demographic variables.

	Total (*n* = 704) mean(sd)	Speech illusion (≥1; n = 145) mean(sd)	No speech illusion (<1; n = 559) mean(sd)
Age in years	17.3(3.5)	16.9(3.6)	17.4(3.4)
Sex			
Female	58.4% (n = 411)	51.7% (n = 75)	60.1% (n = 336)
Male	41.6% (n = 293)	48.3% (n = 70)	39.9% (n = 223)
Zygosity			
Monozygotic	37.4% (n = 263)	33.8% (n = 49)	38.3% (n = 214)
Dizygotic	62.6% (n = 441)	66.2% (n = 96)	61.7% (n = 345)
Education level			
0	0%	0%	0%
1	0.1% (n = 1)	0%	0.2% (n = 1)
2	0%	0%	0%
3	5.5% (n = 39)	4.8% (n = 7)	5.7% (n = 32)
4	10.8% (n = 76)	13.1% (n = 19)	10.2% (n = 57)
5	56.2% (n = 396)	56.6% (n = 82)	56.2% (n = 314)
6	14.2% (n = 100)	14.5% (n = 21)	14.1% (n = 79)
7	10.8% (n = 76)	5.5% (n = 8)	12.2% (n = 68)
Missing	2.3% (n = 16)	5.5% (n = 8)	1.4% (n = 8)
SIS-R			
Positive dimension	0.8(0.4)	0.7(0.3)	0.8(0.4)
Negative dimension	0.4(0.2)	0.4(0.2)	0.4(0.2)
CAPE			
Positive dimension	1.4(0.3)	1.4(0.3)	1.4(0.3)
Negative dimension	1.7(0.4)	1.6(0.3)	1.7(0.4)
Depressive dimension	1.8(0.4)	1.7(0.3)	1.8(0.4)

Speech illusions calculated according to Galdos et al. Education levels range from lower education to university degree; SIS-R: The Structured Interview for Schizotypy—Revised (SIS-R), CAPE: The Community Assessment of Psychic Experiences.

### Measurements

#### The structured Interview for Schizotypy—Revised

The Structured Interview for Schizotypy—Revised (SIS-R) was applied to assess various schizotypy symptoms and signs. The scale has a positive dimension, which includes the symptoms of magical ideation, illusions, referential thinking (2 items), psychotic symptoms, and suspiciousness (6 items), as well as a negative dimension, which includes the symptoms of introversion, social isolation, restricted affect, and poverty of speech (4 items). In total, both dimensions contain 10 items which are scored on a 4-point Likert scale ranging from absent (0) to severe (3) [17].

#### The Community Assessment of Psychic Experiences

The Community Assessment of Psychic Experiences (CAPE) measures positive and negative psychotic experiences and depressive symptoms in nonclinical samples [18]. The questionnaire consists of 42 items (20 items on positive psychotic experiences, 14 items on negative psychotic experiences, and 8 items on depressive feelings) and has two levels measuring the frequency (0  =  never to 4  =  nearly always) and associated distress (1  =  not distressed to 4  =  very distressed) of the different dimensions. The CAPE shows good reliability and validity in general population samples [18, 19]. Data pertaining to the CAPE were collected online.

#### White noise task

The white noise task was developed by Galdos et al. [11] to assess variations in detecting affectively meaningful speech (speech illusion) in neutral random signals (white noise). While wearing earphones, participants listened to three types of stimuli presented randomly across 75 trials: a) white noise only, b) white noise + clearly audible neutral speech, and c) white noise + barely audible neutral speech. The white noise only condition was the main condition of interest. The latter two conditions with audible speech were added to increase expectation, impacting top-down processing. Participants had to rate the 25 trials in each of the three conditions by pressing one of five buttons: 1 = positive speech illusion (endorsed hearing voice with positive valence), 2 = negative speech illusion (endorsed hearing voice with negative valence), 3 = neutral speech illusion (endorsed hearing voice with neutral valence), 4 = no speech heard, and 5 = uncertain (endorsed hearing voice, uncertain affective connotation). The last option aimed to make the rating of 1–3 more conservative. The white noise task was applied with the help of the software E-prime 1.1 (Psychology Software Tools, Pittsburgh, Pennsylvania) and took approximately 15 min. A dichotomized score of “any speech illusion” was calculated, including the occurrence of one or more positive, negative or neutral perceived speech illusion in the ‘white noise only’ condition [11]. Similar to the study by Catalan et al. [10], a second more conservative score was also calculated, which included two or more perceived speech illusion of positive, negative, neutral or uncertain valence. Finally, in accordance to the two methods, scores representing “affectively salient speech illusion” (speech illusion with positive or negative valence) were computed.

### Statistical analysis

Individuals from MZ and DZ twin pairs, with complete data on SIS-R, the white noise task, age and sex were included in the analyses. Age, sex, and education level were assessed as covariates. As the data have a hierarchical organization, individuals were clustered within twin pairs. Statistical analyses were carried out using the Stata, version 13.1 [20] and multilevel logistic regression models were applied using the Stata MEQRLOGIT function.

In an attempt to replicate previous findings as accurately as possible, the methodology of the current study was first construed in line with Galdos et al. [11], and subsequently, the data were also analyzed following the methodology of Catalan et al. [10].

Conform the description in Galdos et al. [11], a more lenient cut-off for any speech illusion (≥1 speech illusion with positive, negative or neutral valence) and affective speech illusion (≥1 speech illusion with positive or negative valence) was used to create the binary speech illusion variables. Furthermore, in order to construct a 4-level schizotypy variable, in line with the previous study, mean scores of the schizotypy variables (positive and negative) were divided into four categories with equal distant values. For ease of interpretation, these categories were recoded (from 0 to 3). Mixed logistic regression analysis was applied using the white noise speech illusion score (any speech illusion and affective speech illusion in separate analyses) as the dependent variable and the schizotypy variable as the independent variable. The analysis was adjusted for age, sex, and educational level.

Consistent with Catalan et al. [10], more conservative binary white noise speech illusion variables were created (any speech illusion: ≥ 2 speech illusion with positive, negative, neutral or uncertain valence; affective speech illusion: ≥ 2 speech illusion with positive or negative valence). Binary measures of both dimensions of the positive schizotypy and the CAPE positive scales were calculated by means of median splits. Multilevel logistic regression models were applied using speech illusion scores as the dependent variables and the binary positive schizotypy and CAPE positive variables as the independent variables. The analyses were adjusted for age and sex.

## Results

An overview of selected demographic variables, the total scores of SIS-R and CAPE, and the distribution of white noise speech illusion [according to 11] is provided in the Table 1.

Of the 704 participants, 145 (20.6%) reported hearing speech in the white noise condition at least once. Education level was missing in 16 participants; therefore, following analyses replicating Galdos et al. [11] were conducted in 688 participants. The replication of Galdos et al. [11] in this sample yielded no association between any speech illusion (with ≥1 speech illusion) and the 4-level variables of schizotypy subscores: the positive schizotypy scale (ORadjusted: 0.82, 95% CI: 0.6–1.12, p = 0.217) and the negative schizotypy scale (ORadjusted: 0.75, 95% CI: 0.56–1.02, p = 0.065). The outcome at the different levels of schizotypy is presented in Table 2: In reference to the lowest level (the level 1), only the level 2 showed a negative association with speech illusion. No significant association was found between positive schizotypy and affectively salient speech illusion (positive and/or negative) (ORadjusted: 0.62, 95% CI: 0.38–1.01, *p* = 0.055).

**Table 2:** White noise speech illusion and the levels of schizotypy.

SIS-R	N	n with any speech illusion	% with any speech illusion	*p*-values	ORadjusted (95% CI)
Positive dimension					
Level 1	269	59	43	------	1
Level 2	369	70	51	0.354	0.83(0.6–1.23)
Level 3	48	8	6	0.394	0.7(0.31–1.59)
Level 4	2	0	0	0.988	------
Negative dimension					
Level 1	208	52	38	------	1
Level 2	395	69	50	0.018	0.61(0.4–0.92)
Level 3	81	15	11	0.185	0.65(0.34–1.2)
Level 4	4	1	1	0.976	------

Speech illusions calculated according to Galdos et al. ORadjusted adjusted for age, sex, education level, and twin pairs; Any speech illusion (≥1) was the dependent variable; 4-level schizotypy was the independent variable (level 1: reference category); SIS-R: The Structured Interview for Schizotypy—Revised (SIS-R).

Using the method by Catalan et al. [10], results indicated that 407 participants perceived no speech illusion (< 2) and 297 (42.2%) participants perceived speech illusion (≥ 2). The replication of Catalan et al. [10] yielded similar results as the method by Galdos et al. [11]. The variable of any speech illusion was not associated with the positive schizotypy variable (ORadjusted: 1.11, 95% CI: 0.79–1.57, *p* = 0.557) or the CAPE positive scale (ORadjusted: 1.25, 95% CI: 0.88–1.79, *p* = 0.220). There was a significant *negative* association between the positive schizotypy variable and illusion with affectively salient valence (positive and/or negative; ORadjusted: 0.44, 95% CI: 0.24–0.81, *p* = 0.008).

## Discussion

The current study investigated the association between speech illusion in the white noise task and subtle psychosis expression in the nonclinical population by replicating the methodology by Catalan et al. [10] and Galdos et al. [11]. Around 20.6% and 42.2% of the participants reported speech illusion perceptions during the white noise task using the methods of Galdos et al. [11] and Catalan et al. [10], respectively. Both rates were higher than the 9.0% and 8.7% found in previous studies [10, 11]. Earlier reports of a positive association between positive schizotypy and the perception of speech illusion could not be replicated in this sample [11], and the current analyses also revealed no association between the experience of speech illusion and the CAPE scores. Thus, in general, the results are in agreement with Catalan et al. [10] showing no significant association between the presence of speech illusion and schizotypy scores in the general population, but contradict the findings of Galdos et al. [11] showing a positive association between positive schizotypy and the perception of speech illusion. Using the method by Galdos et al. [11], there was a significant association between negative schizotypy and any speech illusion between two of the four levels. However, as the general finding was nonsignificant and the different levels consisted of very small subsamples, this was likely a random finding. Analyses of speech illusion with affective valence indicated that the experience of affectively salient speech illusion was associated with lower positive schizotypy scores; however, this only applied to the method by Catalan et al. [10].

An important aspect of the current study was the use of the two analytical methodologies. As Catalan et al. [10] already argued, instead of using a median split, statistical power might be increased by splitting participants’ schizotypy scores according to the highest and lowest values, similar to the method by Galdos et al. [11]. Furthermore, Catalan et al. [10] and Galdos et al. [11] used different cut-off scores and approaches to calculating the dichotomized white noise speech illusion score, which may negatively impact statistical power and speech illusion prevalence rates. Although the present study used both analytical methods, no significant associations between subclinical psychotic symptoms and any speech illusion were observed using either method [10, 11]. This supports the notion that there may be no association between speech illusion during the white noise task and subclinical expressions of positive psychotic experiences.

Although several factors might have influenced the current results, one could interpret these results as suggestive that the mechanisms underlying hallucinations in clinical and nonclinical populations are likely different. It was theorized that hallucinations are caused by a top-down and bottom-up imbalance during the perception process. In clinical samples, an alteration of the top-down processes may mediate psychotic symptoms. However, the current findings suggest that altered top-down processing of sensory information during the white noise task are unrelated to the expression of subclinical symptoms. Thus, speech illusion in the general population, with the possible exception of children [14], may not signal a risk to develop a psychotic disorder and may be subject to different mechanisms compared to clinical samples [10]. Previous work suggests that the affective valence is an important factor discriminating auditory hallucinations in healthy controls and patients [21, 22].

Speech illusion with affective connotation were found to display a strong positive association with psychotic disorders [14, 23]. However, the current results showed that affective salient speech illusion were negatively associated with positive schizotypy—although this was only apparent using the Catalan et al. [10] method. There are several possible explanations for this finding. The first is that it represents a method-related random finding. Another explanation is that the negative association does not necessarily imply that participants with high positive schizotypy scores had fewer illusion with emotional valence; it could also mean that participants with higher scores experienced difficulties in judging the emotional valence of illusion. Previous work indicates that patients with a diagnosis of schizophrenia with auditory-verbal hallucinations, display auditory affect deficits [24, 25]. Another explanation is that the association was confounded by cognitive functioning, as this was not controlled for and cognitive functioning has been suggested to impact affect recognition [26].

Future research may further investigate the significance of cognitive mechanisms, phenomenology, and especially affective valence of speech illusion to better understand the differences between nonclinical and clinical hallucinations. Furthermore, as unique and shared genetic and environmental factors are probably contributing to these differences [3, 4], it may additionally be productive to explore nonclinical hallucinations in relation to genetic and environmental risk factors.

In addition to the theoretical implications, there are several methodological aspects that need to be considered while interpreting the results. In previous studies, attention was mainly directed towards clinical hallucinations, and sample sizes of healthy controls were relatively small. The main strength of the current study is the use of a large general population twin sample. However, splitting the schizotypy scores into four categories, conforming to Galdos et al. [11], resulted in subgroups with very few number of participants, particularly higher risk categories. Therefore, this methodology requires an even larger samples size. Another aspect that needs to be considered is the age range of the different studies. Catalan et al. [10] argued that their sample included participants with a higher mean age than the sample of Galdos et al. [11] and that this could have resulted in differences. This argument was not supported by the current study. The sample had a similar mean age than that of Galdos et al. [11], but the current study could not find an association between speech illusion and positive psychotic experiences. Furthermore, a recent study in children only showed an association between psychotic experiences and speech illusion when the valence of illusion was considered [14]. Finally, an important difference between the previous and the current study is the ethnicity of the samples. The replicated studies were conducted only in the Spanish population; whereas the current study used participants from the Belgium population. It is possible that cultural and language differences contributed to differential findings.

In summary, the results suggest that, in contrast to clinical samples, white noise speech illusion in nonclinical samples are not associated with the expression of subclinical psychotic experiences [10]. On this basis, it can be speculated that the neuropsychological mechanisms underlying the formation of perceptual abnormalities (speech illusion during white noise task) may be different for psychotic patients and nonclinical general population. Therefore, it may be productive for researchers to evaluate differences in cognitive mechanisms, phenomenology, and affective valence across the spectrum. Finally, the results need to be considered with caution as the samples showed important demographic differences, especially in regard to ethnicity and age. Future studies should replicate the current study with increased attention on the impact of demographic confounders. We plan on replicating this study in an even larger database, the European network of national schizophrenia networks studying gene-environment interaction (EU-GEI, see www.eu-gei.eu), which includes heterogeneous, international, multi-ethnic samples of patients, relatives and healthy controls [27, 28].

## Supporting information

S1 Dataset Supporting material: Dataset and labels of education levels. (XLS)
